# Reliability and validity of the Chinese version of the Psychological Climate Scale among nuclear emergency responders

**DOI:** 10.3389/fpsyg.2026.1805085

**Published:** 2026-04-01

**Authors:** Li Peiran, Bai Jie, Guo Yangyang, Hao Subing, Chen Jie

**Affiliations:** 1 School of Medicine, Yangtze University, Jingzhou, China; 2 Department of Quality Management, Rocket Force Characteristic Medical Center of the PLA, Beijing, China; 3 Department of Respiratory Medicine, Rocket Force Characteristic Medical Center of the PLA, Beijing, China; 4 Outpatient Department, Rocket Force Characteristic Medical Center of the PLA, Beijing, China; 5 Department of Psychology, Tsinghua University, Beijing, China

**Keywords:** nuclear emergency response, psychological climate, reliability, scale, validity

## Abstract

**Objective:**

To translate and culturally adapt the Psychological Climate Scale into Chinese and to examine its reliability and validity among nuclear emergency responders, thereby providing a reliable instrument for assessing their organizational psychological climate.

**Methods:**

The Brislin translation–back-translation model was employed to translate and culturally adapt the Psychological Climate Scale. Following expert consultation and a pilot study, a Chinese version of the scale was developed. A total of 286 nuclear emergency responders from three provinces (Beijing, Hunan, and Yunnan) were recruited to evaluate the reliability and validity of the scale.

**Results:**

The final Chinese version comprised 18 items across two dimensions. The overall Cronbach’s *α* coefficient was 0.953, with *α* values of 0.944 and 0.929 for “Supervisor Support and Work Value” and “Organizational Constraints and Emotional Expression,” respectively. The split-half reliability was 0.951, and the two-week test–retest reliability coefficient was 0.782. Content validity was satisfactory (I-CVI: 0.900–1.000; S-CVI/Ave: 0.953). Exploratory factor analysis supported a two-factor structure (KMO = 0.945), explaining 68.267% of the total variance.

**Conclusion:**

The Chinese version of the Psychological Climate Scale demonstrated good reliability and validity among nuclear emergency responders. The instrument provides a useful tool for assessing psychological climate in this population and may offer an empirical basis for organizational support strategies and targeted psychological interventions.

## 1 Introduction

The development of nuclear energy, while promoting socio-economic progress, is accompanied by the inherent risk of nuclear safety incidents (Li and Zhang, 2017). Nuclear emergency response represents a critical component in managing nuclear and radiological emergencies and is characterized by sudden onset, complex on-site operations, stringent professional protection requirements, and profound psychosocial impacts (Carr et al., 2018; Loganovsky and Marazziti, 2021). As frontline operators, nuclear emergency responders—who work long-term in nuclear-related environments and participate in regular emergency training—are exposed to sustained high-pressure and high-risk working conditions (Rebmann et al., 2019; Petrovic et al., 2026). Their psychological status is closely linked to organizational support, which not only affects the quality of emergency operations but also directly influences their physical and mental health, as well as the long-term sustainability of the response workforce (Xiao et al., 2020; Centre for Nuclear and Radiation Safety, Ministry of Ecology and Environment, 2024). They undergo regular intensive training through efficient teamwork, leadership, continuous learning, and strong commitment and work engagement. They also endure prolonged psychological pressure stemming from uncertainty threats and demanding skill requirements. This implies that individual capability and motivation alone cannot guarantee mission success; reliance on the context and support provided by the organization is essential. Consequently, the organizational environment significantly influences both personnel and organizational overall effectiveness and long-term sustainability (Chen et al., 2022).

Psychological climate refers to individuals’ subjective perceptions and interpretations of their organizational environment, practices, and their personal meaning (James et al., 2008). Existing research on specialized occupational groups has predominantly focused on organizational climate, which emphasizes shared perceptions of organizational policies and work environment characteristics at the collective level (Atitsogbui and Amponsah-Tawiah, 2019). In contrast, psychological climate focuses on employees’ individual-level perceptions and interpretations of the work environment, which may vary considerably depending on occupational context, personal experiences, and psychological states, reflecting substantial inter-individual variability (Singh, 2025). Thus, while organizational climate represents a collective, group-level construct, psychological climate reflects employees’ subjective experiences at the individual level. Nuclear emergency response work is characterized by high risk, high stress, and a high degree of collaboration. Responders are required to rapidly execute complex tasks under conditions of uncertainty and potential threat. In this context, psychological climate is not only closely related to individual mental wellbeing but also directly affects the accuracy of emergency operations and the efficiency of team coordination (Bazaluk et al., 2024). Therefore, the development of a scientifically sound assessment tool for the systematic and long-term evaluation of psychological climate among nuclear emergency response teams is of substantial practical significance. Such assessment provides an empirical basis for organizational support strategies and targeted psychological interventions.

Currently, psychological climate assessment instruments used in related fields in China are predominantly derived from organizational climate frameworks (Wu et al., 2025; Jie and Wang, 2023; Lin, 2019), with limited availability of tools specifically designed to capture the distinctive features of psychological climate. As a result, these instruments may not fully accommodate the unique operational contexts and psychological demands of nuclear emergency response work. The localization and validation of internationally established and psychometrically robust instruments can enhance both the scientific rigor and cross-cultural comparability of assessments.

Brown and Leigh (1996) developed the Psychological Climate Scale based on cognitive appraisal theory, providing a multidimensional framework for assessing individuals’ perceptions of organizational psychological environments. To date, no validated Chinese version of this scale has been reported. Therefore, the present study aimed to translate and culturally adapt the Psychological Climate Scale and to examine its reliability and validity among nuclear emergency responders, thereby providing a reliable assessment tool for this high-risk occupational group.

## 2 Localization of the scale

### 2.1 Introduction to the scale

Based on the cognitive appraisal theory of psychological climate proposed by James and Jones (1974) and informed by Kahn’s ethnographic research on work engagement (Kahn, 1992), Brown and Leigh developed the Psychological Climate Scale. The scale is designed to assess employees’ subjective perceptions of psychological safety within their work environment and to systematically evaluate perceptions of organizational context, managerial practices, and work atmosphere.

The original scale consists of six dimensions comprising 21 items and is rated on a seven-point Likert scale ranging from 1 (“strongly disagree”) to 7 (“strongly agree”). Higher total scores indicate a more positive perceived psychological climate. The six dimensions include supportive management (5 items), role clarity (3 items), work contribution (4 items), work recognition (3 items), self-expression (4 items), and work challenge (2 items). The scale has demonstrated satisfactory reliability and validity in previous cross-cultural adaptations, including a Turkish version published in 2017 (Argon and Limon, 2017).

### 2.2 Bidirectional translation of the scale

This study followed Brislin’s translation–back-translation model to culturally adapt the original English-language Psychological Climate Scale into Chinese (Jones et al., 2001). Attempts were made to contact the original authors through academic databases and institutional websites; however, no response was received. The adapted scale is intended solely for academic research use.

The translation process comprised four stages. First, two independent bilingual translators conducted forward translations of the original scale into Chinese. One translator was a medical doctoral researcher with overseas study experience, and the other was a master’s-level nursing student with advanced English proficiency. Second, a third researcher who was not involved in the initial translation synthesized the two versions, followed by group discussion among translators and the research team to resolve discrepancies and achieve consensus, resulting in the preliminary Chinese version (Version A).

Third, two additional translators with no prior exposure to the original scale independently back-translated Version A into English. One translator, a postgraduate student in English linguistics, focused on linguistic accuracy, while the other, a doctoral student with overseas training in psychology, emphasized conceptual equivalence. These back-translations were compared and consolidated into a single version (Version B). Finally, Version B was reviewed alongside the original scale by two experts with experience in emergency nursing research and psychological intervention. Semantic equivalence, cultural appropriateness, and contextual relevance were evaluated item by item, leading to the development of the preliminary Chinese draft (Version C).

### 2.3 Cultural debugging

To ensure the cultural appropriateness and content validity of the Chinese version of the Psychological Climate Scale, a two-round expert consultation was conducted with 10 specialists. The experts were recruited from the fields of emergency psychology, medical and nursing psychology, occupational mental health, and scale development. All experts held associate senior professional titles or above and had more than 10 years of relevant professional experience.

During each round of consultation, experts independently evaluated each item using a four-point Likert scale with respect to relevance, clarity of expression, importance, and cultural suitability within the Chinese context. Space was also provided for qualitative comments and specific revision suggestions. Following the first round, items were revised by the research team based on quantitative ratings and qualitative feedback. The revised version was then redistributed for the second round of consultation to achieve consensus.

Based on expert feedback across both rounds, item wording and expressions were refined to enhance semantic clarity, cultural equivalence, and contextual relevance for nuclear emergency response settings. The resulting version was used for the subsequent pilot study.

### 2.4 Pilot study

Prior to the formal investigation, a pilot study was conducted to evaluate the clarity, comprehensibility, and cultural appropriateness of the preliminary Chinese version of the Psychological Climate Scale. A convenience sample of 30 nuclear emergency responders was recruited for this purpose.

All procedures involving human participants, including the pilot study and the formal survey, were approved by the Ethics Committee of the authors’ affiliated institution (Approval No. KY2026001). Written informed consent was obtained from all participants prior to data collection. Based on participants’ feedback and discussions within the research team, minor revisions were made to several items to improve clarity and contextual relevance. Data obtained from the pilot study were not included in the final statistical analyses.

## 3 Reliability and validity testing of the scale

### 3.1 Sample

Convenience sampling was employed to recruit nuclear emergency rescue personnel from three provinces—Beijing, Hunan, and Yunnan—between September and November 2025. Inclusion and exclusion criteria were consistent with those used in the preliminary survey. Exploratory factor analysis generally requires a sample size of 5–10 times the number of items. The initial scale comprised 21 items. Based on a calculation of five times the number of items and allowing for a 20–30% rate of invalid responses, the estimated sample size ranged from 132 to 150 (Wu, 2010; Costello and Osborne, 2005). Accordingly, the minimum required sample size for this study was determined to be 132 respondents. The actual number of valid questionnaires collected was 286, which far exceeded the minimum requirement and provided sufficient statistical power for exploratory factor analysis. Furthermore, 30 participants were randomly selected from the valid sample for retesting 2 weeks after the initial survey. Pearson’s correlation analysis was used to calculate the test–retest reliability coefficient, thereby evaluating the stability of the scale’s measurement outcomes.

### 3.2 Survey instruments and data collection

The survey instruments included the Chinese version of the Psychological Climate Scale and a researcher-designed general information questionnaire. The latter was developed through a systematic literature review and research group discussions to collect participants’ sociodemographic data. The General Information Questionnaire covered gender, age, length of service, educational attainment, job role, and marital and family status. With the support of relevant collaborating units, personnel who were not on duty were centrally assembled by department or work team. Following the acquisition of informed consent from participants, the questionnaire was administered anonymously. Prior to questionnaire distribution, research team members who had undergone standardized training provided participants with detailed explanations regarding the survey’s purpose, significance, confidentiality, and completion procedures. The questionnaire required approximately 10–20 min to complete. Questionnaires were collected on site and preliminarily checked for completeness, with any missing items addressed immediately. A total of 300 questionnaires were distributed for this study, yielding 286 valid responses, representing a valid response rate of 95.3%.

### 3.3 Statistical methods

Data entry and organization were performed using Excel 2021. Statistical analyses were conducted using SPSS version 24.0 for item analysis, reliability analysis, descriptive statistics, and exploratory factor analysis (EFA). Confirmatory factor analysis (CFA) and bootstrap procedures were conducted using IBM SPSS Amos version 24.0. Parallel analysis and visualization of the scree plot were performed using Python. Because of the limited sample size, EFA and CFA were conducted using the same dataset. To reduce potential overfitting and improve the robustness of the model estimates, bootstrap resampling (2,000 samples) was performed in AMOS to obtain bias-corrected estimates and confidence intervals. Criterion-related validity was evaluated by examining the correlations between the Psychological Climate Scale scores (including the total score and factor scores) and mental health status measured by the Chinese Military Mental Health Scale (CMMHS). Pearson correlation analysis was used to assess the associations between these variables. The significance level *α* was set at 0.05. For descriptive statistics, demographic data and other categorical variables were presented as frequencies and percentages. Continuous variables were assessed for normality using the Shapiro–Wilk test; variables with a normal distribution were expressed as mean ± standard deviation.

#### 3.3.1 Item analysis method

Item analysis was first conducted to screen scale items, specifically using the critical ratio method and the correlation coefficient method to evaluate each item’s discriminative power and representativeness (Du, 2018). (1) Critical Ratio Method: Participants in the top 27% of total scale scores were assigned to the high-score group, and those in the bottom 27% to the low-score group. An independent samples *t*-test was used to compare score differences between groups on each item. Items showing no statistically significant difference between groups (*p* > 0.05) or a critical ratio value less than 3.0 were considered to have insufficient discrimination and were considered for deletion. (2) Internal consistency principles were also applied during item screening. If deletion of an item resulted in an increase in the overall Cronbach’s *α* coefficient, the item was considered detrimental to internal consistency and subject to removal.

#### 3.3.2 Reliability analysis

Cronbach’s *α* coefficient was used to assess the internal consistency of the scale, and split-half reliability was used to test item consistency using an odd–even split. Thirty participants were selected for retesting after 2 weeks. A paired samples *t*-test was first conducted to examine differences in total scale and dimension scores between the two measurements. Subsequently, Pearson correlation analysis was conducted to calculate the test–retest reliability coefficient and evaluate measurement stability. Cronbach’s *α* coefficient > 0.80 and a test–retest reliability coefficient > 0.70 were considered indicative of good reliability (American Educational Research Association et al., 1999).

#### 3.3.3 Validity analysis

Ten experts in relevant fields were invited to evaluate content validity using a four-point Likert scale. Item-level content validity indices (I-CVI) and the scale-level average content validity index (S-CVI/Ave) were calculated. Content validity was considered satisfactory when I-CVI ≥ 0.78 and S-CVI/Ave ≥ 0.90 (Kaiser, 1970). The Kaiser–Meyer–Olkin (KMO) test and Bartlett’s test of sphericity were used to assess the suitability for factor analysis. Common factors were extracted using principal component analysis with varimax orthogonal rotation. Factors with eigenvalues > 1 were retained, and structural validity was considered satisfactory when the cumulative variance explained was ≥60% and item factor loadings were >0.50 (American Educational Research Association et al., 1999). Parallel analysis for factor retention was performed using Python.

#### 3.3.4 Confirmatory factor analysis and bootstrap validation

CFA was conducted using AMOS version 24.0 to test the factor structure identified in the EFA. Due to the limited sample size, the same dataset was used for both analyses. Maximum likelihood estimation was applied to estimate model parameters. Model fit was evaluated using multiple fit indices, including the chi-square statistic divided by degrees of freedom (*χ*^2^/df), the Comparative Fit Index (CFI), the Tucker–Lewis Index (TLI), the Root Mean Square Error of Approximation (RMSEA), and the Root Mean Square Residual (RMR). Acceptable model fit was defined as *χ*^2^/df < 3, CFI ≥ 0.90, TLI ≥ 0.90, RMSEA ≤ 0.08, and RMR ≤ 0.08 (Hu and Bentler, 1999).

To further examine the stability and robustness of the model, bootstrap resampling was performed with 2000 samples. The bootstrap procedure was used to evaluate the stability of parameter estimates and the overall model fit across repeated samples. Stable parameter estimates and consistent model fit across bootstrap samples were considered evidence supporting the robustness of the factor structure.

#### 3.3.5 Criterion-related validity

Criterion-related validity was examined by analyzing the relationship between psychological climate and mental health status. Psychological climate reflects employees’ perceptions of their work environment, including supervisory support, organizational constraints, and the value attached to work. Previous research in organizational psychology has demonstrated that psychological climate is closely associated with employees’ psychological wellbeing and mental health outcomes (Parker et al., 2003). In high-risk occupational settings such as emergency response work, organizational support and social support have been shown to significantly influence psychological distress and mental health among responders (Matsuoka et al., 2012). Therefore, mental health status was selected as an external criterion variable.

Mental health status was assessed using the Chinese Military Mental Health Scale (CMMHS), which has demonstrated good reliability and validity for assessing psychological symptoms among Chinese populations (Wang et al., 2012). Although originally developed for military personnel, the scale evaluates general psychological health symptoms rather than occupation-specific characteristics. Therefore, it can be applied to other high-demand occupational groups exposed to substantial work-related stress, such as nuclear emergency responders (Carr et al., 2018; Loganovsky and Marazziti, 2021).

## 4 Results

### 4.1 Cultural commissioning and pre-survey results

**Figure 1:**
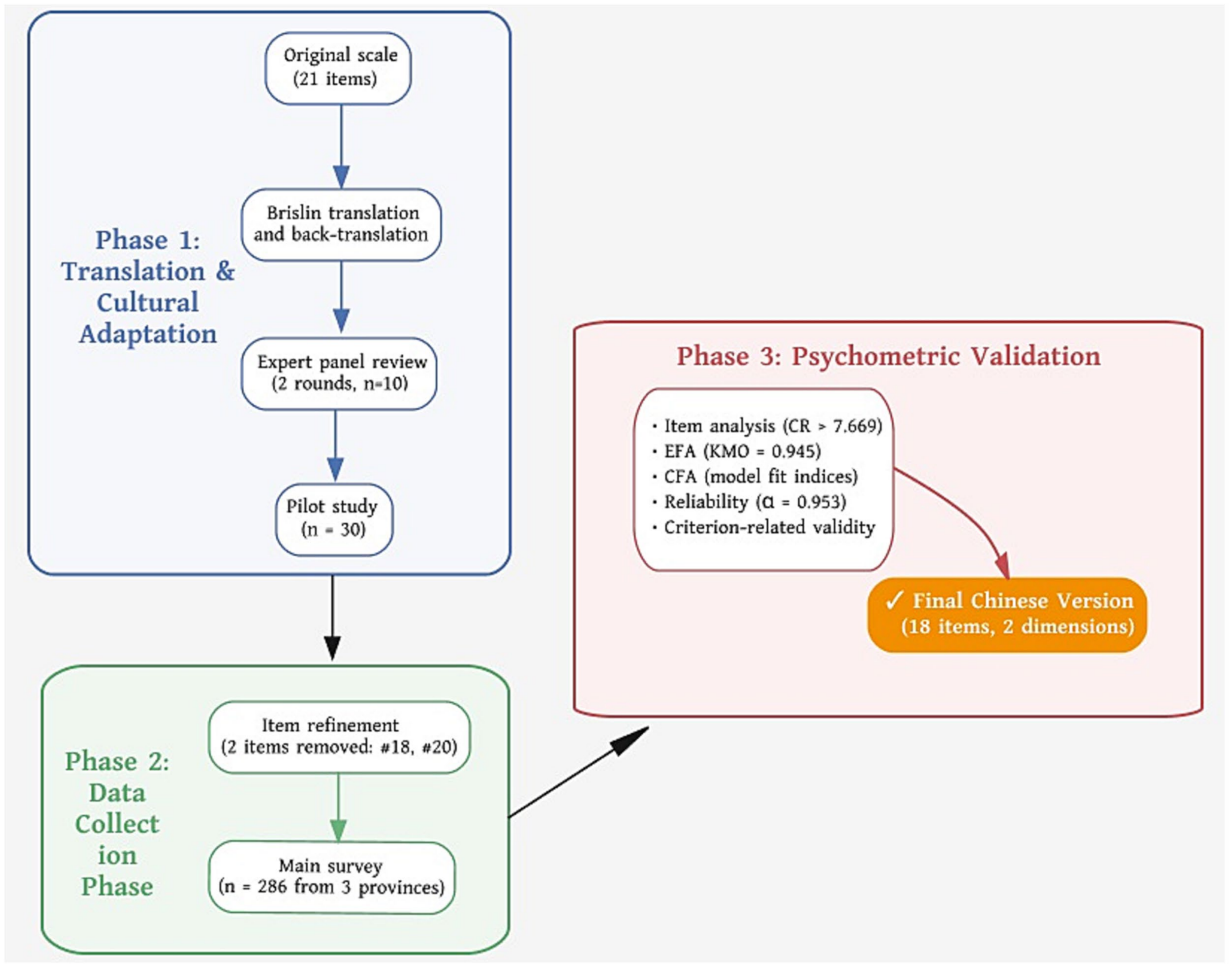
Translation and validation process of the Chinese Psychological Climate Scale.

The process of translating, adapting, and validating the Chinese version of the Psychological Climate Scale is illustrated in Figure 1. It involved forward-back translation, expert review, a pilot study, and psychometric evaluation, resulting in an 18-item instrument with two dimensions.

During the cross-cultural adaptation of the scale, individual items were appropriately adjusted based on translation and cultural adaptation principles, incorporating expert evaluations and pre-test feedback. (1) Item 20, “My work is highly challenging,” was removed. Nuclear emergency response constitutes a high-risk and high-pressure occupational context in which tasks are inherently demanding. During the pre-test, most participants demonstrated similar understanding and evaluation of this item, making it less effective in distinguishing individual differences in perceptions of the organizational environment. Following discussions between the research team and domain experts, this item was excluded. (2) Item 18, “I cannot freely express my true self at work,” was a reverse-scored item. Pre-test feedback indicated that some participants experienced difficulty understanding this statement. In the highly disciplined and task-oriented environment of emergency response organizations, this item could lead to interpretation bias and potentially affect response consistency. After comprehensive consideration of participant feedback and expert opinions, this item was removed.

Throughout the scale revision process, the revision was guided by the theoretical framework of psychological climate. Through expert review, the item content and conceptual dimensions were carefully evaluated to ensure that the retained items adequately covered the core conceptual domains addressed by the original scale. This study only removed or adjusted individual items without altering the core dimensions of the scale. Therefore, the primary conceptual structure of the scale remains consistent with that of the original version. The resulting Chinese version mainly involves moderate simplification at the item level to improve cultural appropriateness and comprehensibility among nuclear emergency response personnel in China, without altering the fundamental theoretical scope of the psychological climate construct.

### 4.2 General information of the respondents

The 286 nuclear emergency responders had a mean age of 24.55 ± 4.61 years and a mean length of service of 4.74 ± 4.81 years; additional demographic characteristics are presented in Table 1.

**Table 1:** General information of nuclear emergency rescue personnel (*n* = 286).

Characteristic		*n*	%
Gender	Male	278	97.20%
	Female	8	2.80%
Education level	Specialist	186	65.03%
	Bachelor’s degree	92	32.17%
	Master’s degree	6	2.10%
	Doctorate	2	0.70%
Only child	Yes	93	32.52%
	No	193	67.48%
Marital status	Unmarried	252	88.11%
	Married	31	10.84%
	Divorced	2	0.70%
	Veuf	1	0.35%

### 4.3 Item analysis results

Item analysis results showed that CR values for all items ranged from 7.669 to 24.157 (*p* < 0.05), indicating significant discriminatory power between the high- and low-score groups. Following reverse-score conversion, Pearson correlation coefficients between all items (including Item 13) and the total scale score ranged from 0.377 to 0.789 (all *p* < 0.05). With the exception of Item 13, correlation coefficients between the remaining items and the total score were all greater than 0.50 (0.571–0.789), indicating that most items showed high consistency with the overall scale content and demonstrated good representativeness and discrimination. Although Item 13 (“I seldom feel my work is taken for granted”) showed a Pearson correlation coefficient of 0.377 with the total score, it was retained due to its unique and theoretical significance in measuring work identification. In addition, its critical ratio (CR = 7.669, *p* < 0.05) indicated satisfactory discriminative power. Overall, all items met the retention criteria, and no items were excluded.

### 4.4 Results of scale validity testing

#### 4.4.1 Content validity

The results showed that the I-CVI of the Chinese version of the Psychological Climate Scale ranged from 0.90 to 1.00, with an S-CVI/Ave of 0.953, indicating good content validity.

#### 4.4.2 Construct validity

##### 4.4.2.1 Exploratory factor analysis (EFA)

The KMO test and Bartlett’s test of sphericity showed that the KMO value of the Chinese version of the Psychological Climate Scale for nuclear emergency responders was 0.945. Bartlett’s test of sphericity yielded a chi-square value of 4684.067 (*p* < 0.001), indicating suitability for factor analysis (see Table 2). Common factors were extracted using principal component analysis with varimax orthogonal rotation. Factors with eigenvalues > 1 were retained, yielding two common factors that explained 68.267% of the cumulative variance. The factor loadings for each item ranged from 0.409 to 0.872. Except for Item 13 (“I rarely feel my work is taken for granted”), which showed a slightly lower loading (0.409), all other items had factor loadings exceeding 0.50, indicating strong associations between the items and their respective factors. Considering the relatively low factor loading and weak overall item–total correlation coefficients observed in the item analysis, Item 13 was ultimately removed (Ledesma and Valero-Mora, 2007; Auerswald and Moshagen, 2019).

**Table 2:** The Chinese version of the Psychological Climate Scale KMO and Bartlett test (*n* = 286).

Items	Inspection criteria	Statistical value
KMO test	Valeur KMO	0.945
Bartlett’s Sphericity test	*χ* ^2^	4,684.067
	df	66
	*p*	<0.001

**Figure 2:**
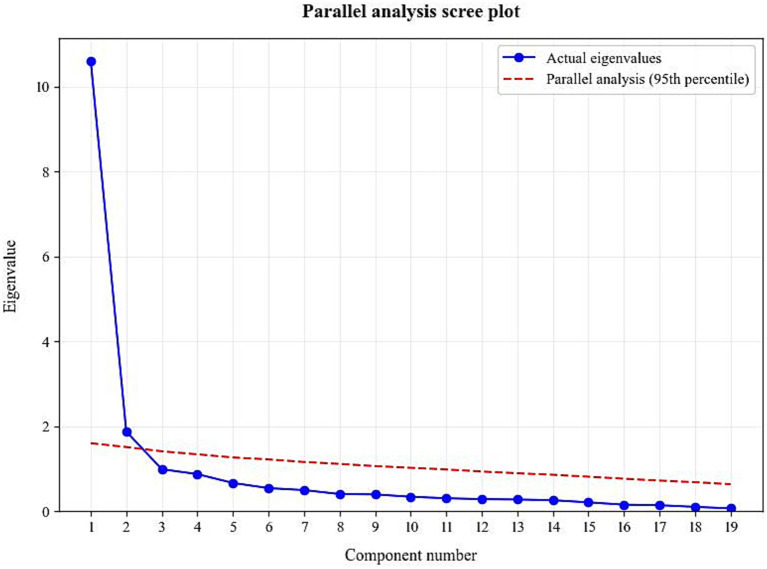
Parallel analysis scree plot. The solid line represents the observed eigenvalues, and the dashed line represents the 95th percentile of eigenvalues obtained from randomly generated data.

To determine the appropriate number of factors to retain, parallel analysis was conducted using Python, and scree plot inspection was performed. As shown in Figure 2, only the first two observed eigenvalues exceeded the 95th percentile of eigenvalues obtained from randomly generated data, supporting the retention of a two-factor solution. Factor 1 comprised items from the original dimensions of “Supportive Management,” “Role Clarity,” “Work Contribution,” and “Work Recognition,” collectively reflecting a supportive and well-structured work environment.

Factors were named based on item content and theoretical constructs. Factor 2 included items from the original dimensions of “Self-Expression” and “Work Challenge,” along with several reverse-scored items, reflecting organizational constraints and emotional expression. Although item assignments diverged from the original scale’s theoretical dimensions, they remained consistent with the core essence of psychological climate. The factor structure was clear and demonstrated strong explanatory power, indicating that the Chinese version of the Psychological Climate Scale exhibited sound construct validity among nuclear emergency response personnel. After adjustment, item loadings within their respective dimensions ranged from 0.544 to 0.849 (see Table 3 for details).

**Table 3:** Rotated factor loading matrix for the Chinese version of the Psychological Climate Scale (*n* = 286).

Items	Superior support and sense of job value	Organizational constraints and emotional expression
Q1	**0.685**	0.373
Q2	**0.741**	0.346
Q3	**0.659**	0.302
Q7	**0.547**	0.313
Q8	**0.830**	0.307
Q9	**0.872**	0.274
Q10	**0.856**	0.271
Q11	**0.772**	0.294
Q14	**0.817**	0.319
Q15	**0.817**	0.307
Q4	0.323	**0.724**
Q5	0.303	**0.784**
Q6	0.382	**0.759**
Q12	0.300	**0.731**
Q16	0.260	**0.700**
Q17	0.247	**0.818**
Q18	0.311	**0.790**
Q19	0.284	**0.772**

Bold values indicate factor loadings greater than 0.50.

##### 4.4.2.2 Confirmatory factor analysis (CFA)

**Figure 3:**
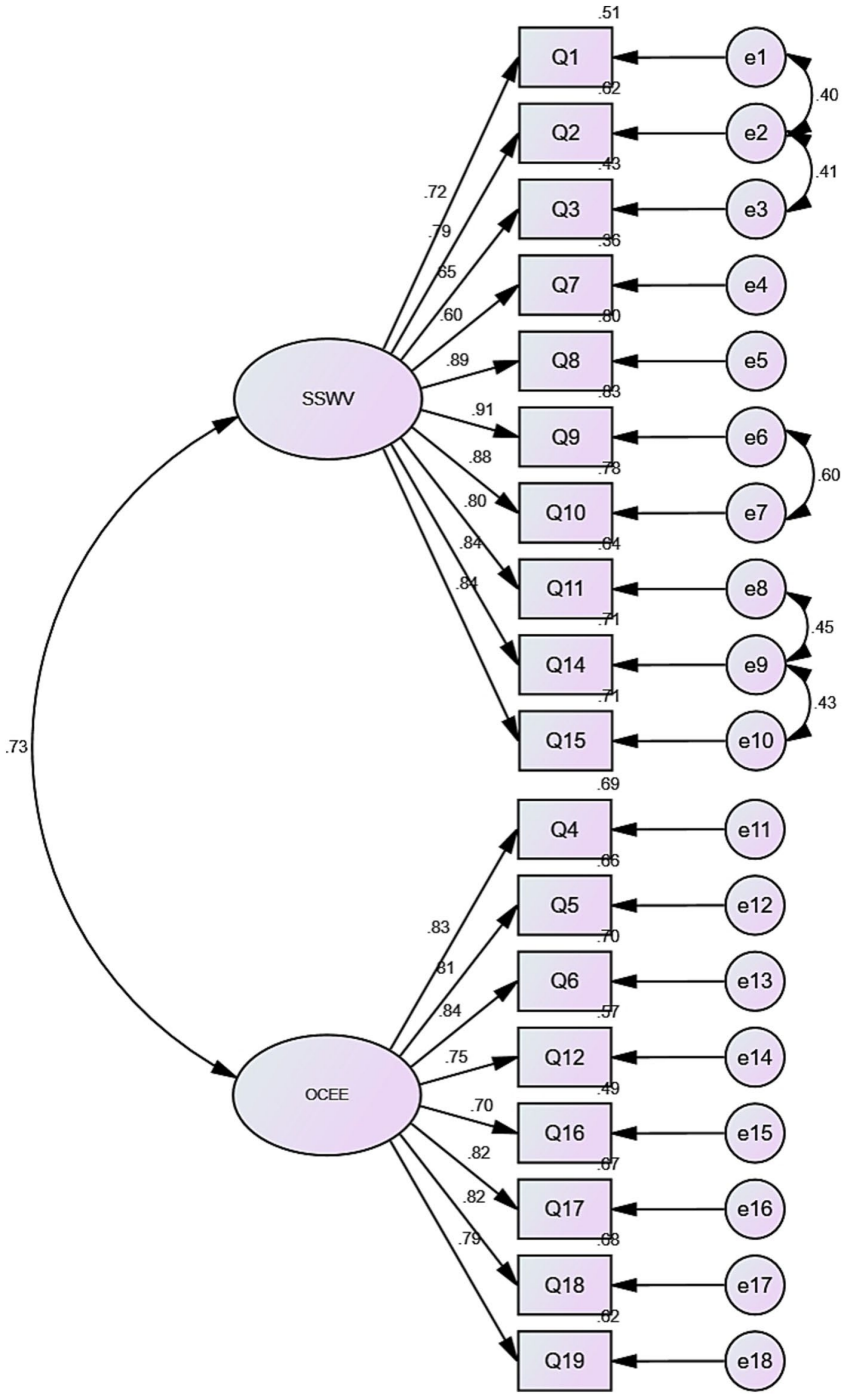
Confirmatory factor analysis model of the Chinese version of the Psychological Climate Scale (standardized estimates). SSW, Superior Support and Sense of Job Value; OCEE, Organizational Constraints and Emotional Expression.

CFA was conducted using AMOS version 24.0 to test the two-factor structure identified in the exploratory factor analysis. The standardized CFA model is presented in Figure 3. The results indicated that the model demonstrated an acceptable fit to the data (*χ*^2^/df = 2.663, CFI = 0.954, TLI = 0.945, RMSEA = 0.076, RMR = 0.062). These indices suggested that the proposed two-factor model adequately represented the underlying structure of the Psychological Climate Scale among nuclear emergency responders.

### 4.5 Reliability test results for the scale

The Chinese version of the Psychological Climate Scale showed an overall Cronbach’s *α* coefficient of 0.953, indicating strong internal consistency. The extracted dimensions—“Superior Support and Sense of Work Value” and “Organizational Constraints and Emotional Expression”—yielded Cronbach’s *α* coefficients of 0.944 and 0.929, respectively. Both dimensions exhibited good reliability, suggesting high homogeneity among items within each scale. After deletion of any item, the overall Cronbach’s *α* coefficient showed no significant improvement, remaining within the range of 0.950–0.953. This indicates that all items contributed stably to the reliability of the total scale, and no items required removal. The split-half reliability coefficient was 0.951. Test–retest reliability analysis revealed no statistically significant difference between the two measurements based on a paired samples *t*-test, indicating the absence of systematic measurement bias. The correlation coefficient between the two measurements was *r* = 0.782 (*p* < 0.05), suggesting good temporal stability of the scale.

### 4.6 Bootstrap validation

To further evaluate the stability of the model, bootstrap resampling with 2,000 samples was conducted. The results showed that the model fit better in all bootstrap samples, with no cases of poorer fit or convergence failure. These findings indicate that the model parameters were stable and robust across repeated resampling.

### 4.7 Criterion-related validity

Criterion-related validity was assessed by examining the correlation between the Psychological Climate Scale and mental health status measured by the CMMHS. Pearson correlation analysis showed that psychological climate was significantly negatively correlated with mental health symptoms (*r* = −0.415, *p* < 0.01). This result indicates that a more positive psychological climate was associated with better mental health status, providing evidence for the criterion-related validity of the scale.

## 5 Discussion

### 5.1 The reliability and validity of the Chinese version of the Psychological Climate Scale is good among nuclear emergency rescue personnel

This study followed Brislin’s translation model to localize the questionnaire, employing standard procedures including forward translation, synthesis, back-translation, and cultural adaptation. Item wording and selection were optimized to align with the occupational characteristics of nuclear emergency responders, thereby preserving the core theoretical constructs of the original scale while enhancing cultural appropriateness and readability. Reliability testing revealed Cronbach’s *α* coefficients exceeding 0.90 for both the total scale and individual dimensions, with a split-half reliability coefficient of 0.951 and a test–retest reliability coefficient of 0.782. These findings suggest that the Chinese version of the Psychological Climate Scale demonstrates acceptable psychometric properties and effectively reflects the psychological climate perceived by nuclear emergency personnel.

The validity of the scale was systematically evaluated through content validity, construct validity, and criterion-related validity. Expert assessment yielded an S-CVI/Ave of 0.953, with all item-level I-CVIs ≥ 0.90, indicating that the scale items effectively captured the core concepts of psychological climate. Exploratory factor analysis extracted two common factors that explained 68.267% of the variance, with generally high factor loadings and a clear structure. Further confirmatory factor analysis showed favorable model fit indices (*χ*^2^/df = 2.663, CFI = 0.954, TLI = 0.945, RMSEA = 0.076), indicating that the two-factor structure adequately explained the latent structure of psychological climate among nuclear emergency responders. Bootstrap results further confirmed the stability of the model parameters.

Additionally, this study examined the criterion-related validity of the scale. The results showed a significant negative correlation between the overall psychological climate score and mental health symptoms (*r* = −0.415, *p* < 0.01), suggesting that individuals who perceived a more positive psychological climate within their organizational environment tended to report better mental health. This finding is consistent with theoretical expectations from organizational psychology research regarding the relationship between psychological climate and employee mental health, further supporting the validity of the scale.

### 5.2 Rationale for scale dimension adjustment

Following the localized revision of the Psychological Climate Scale, this study ultimately established a two-factor structure. Compared with the multidimensional structure of the original scale, this outcome reflects the integration of several dimensions. Such structural differences do not necessarily indicate a simplification of the scale; rather, they may reflect variations in how individuals perceive organizational environments across different cultural contexts. Psychological climate theory posits that psychological climate results from individuals constructing meaning about work situations through social cognitive processes (James et al., 2008). Due to differences in cultural backgrounds, organizational structures, and work contexts, different groups may form distinct cognitive dimensional structures when perceiving and organizing environmental information. Cross-cultural psychometric research indicates that during the cross-cultural adaptation of scales, the original dimensions may undergo integration, differentiation, or reorganization, which is typically driven by changes in cognitive structures resulting from differences in cultural contexts and work situations (Berry et al., 2010).

The two factors extracted in this study are consistent with the theoretical framework of psychological climate. The first factor, “Superior Support and Sense of Job Value,” primarily integrates dimensions such as supportive management, role clarity, work contribution, and work recognition from the original scale. Theoretically, these dimensions reflect whether the organization provides a supportive environment and whether employees perceive the meaning and value of their work. Related research indicates that leadership support not only influences employees’ evaluations of the organizational environment but also significantly enhances their sense of meaning and value in their work (Li et al., 2025; Zhang et al., 2025). Therefore, integrating these dimensions into a broader “organizational support–work meaning” construct has theoretical validity within specific occupational groups.

The second factor, “Organizational Constraints and Emotional Expression,” primarily reflects individuals’ perceptions of freedom of expression and work stress within organizational settings. Psychological climate theory emphasizes that employees’ psychological safety and opportunities for expression at work are crucial factors influencing behavior and emotional responses (Kahn, 1992). In high-risk occupational settings, organizational norms and task pressures are often intense, which may heighten employees’ sensitivity to whether the organization allows the expression of genuine emotions or opinions. Consequently, items related to self-expression statistically formed an independent factor, reflecting the interactive relationship between organizational constraints and emotional expression.

Occupational contextual factors may contribute to the integration of these dimensions. The nature of nuclear emergency response work requires personnel to rapidly execute tasks in complex environments while maintaining high levels of organizational discipline. Within such task-oriented organizations, employees’ perceptions of the organizational environment typically focus on two core aspects: whether the organization provides sufficient support and meaning in work, and whether organizational rules and task requirements impose constraints on individual expression and emotions. Similar patterns have been observed in other emergency or high-stress occupational groups. For instance, studies on disaster responders and trauma-exposed populations indicate that organizational support and opportunities for emotional expression are critical environmental factors influencing psychological adaptation and mental health (Lang et al., 2003; Pietrzak et al., 2014; She et al., 2022). Therefore, the two-factor structure identified in this study may better reflect the practical operational context of nuclear emergency responders.

Furthermore, Chinese organizational culture generally emphasizes teamwork, hierarchical structures, and organizational belonging. These cultural characteristics may further reinforce employees’ overall perceptions of two core environmental dimensions: “organizational support” and “organizational constraints.” Therefore, the two-factor structure identified in this study not only reflects the occupational characteristics of nuclear emergency response work but may also represent distinctive patterns of psychological climate perception within the context of Chinese organizational culture.

In summary, although the factor structure identified in this study differs somewhat from that of the original scale, it still captures the core concepts of psychological climate theory and demonstrates strong explanatory power within the specialized occupational group of nuclear emergency responders.

### 5.3 Practical implications of the scale

This study expands the application of psychological climate theory to high-risk emergency response contexts. Previous research on psychological climate has primarily focused on organizational contexts such as corporate employees, healthcare workers, and educators (Espinoza-Díaz et al., 2023; Weziak-Bialowolska et al., 2023). However, high-risk occupational organizations often differ substantially in task stress, organizational structure, and decision-making processes, which may influence employees’ perceptions of the organizational environment. In this study, the Psychological Climate Scale was validated among nuclear emergency responders, and the results indicated a certain degree of dimensional integration within the factor structure. These findings suggest that in highly regulated and task-oriented organizational contexts, employees’ perceptions of the organizational environment may be concentrated on several core dimensions rather than on multiple relatively independent environmental characteristics. This further indicates that the structure of psychological climate may be influenced by occupational contextual factors and may not be entirely consistent across different organizational environments.

The adapted scale provides a specialized tool for assessing the organizational psychological environment of China’s nuclear emergency response teams. Compared to general organizational climate scales, it more accurately identifies the psychological perceptions of rescue personnel in high-risk environments. This provides empirical evidence to support managerial efforts to optimize support mechanisms, clarify operational procedures, and strengthen team communication. Through regular assessments, organizations may identify management strengths and potential risks. This can enhance frontline support, optimize task allocation, and promote targeted team communication. By conducting regular psychological climate assessments, organizational managers can systematically identify key organizational factors influencing the psychological experiences of rescue personnel. This enables the optimization of management strategies, such as strengthening leadership support, improving communication mechanisms, and establishing more robust psychological support systems. In summary, psychological climate assessments not only contribute to enhancing the mental health of rescue personnel but may also further promote team collaboration efficiency and the quality of emergency task execution (Weziak-Bialowolska et al., 2020; Yuan et al., 2024).

Moreover, although the present study focused on nuclear emergency responders, the scale may also have potential applicability to other high-risk occupational groups, such as firefighters and emergency medical personnel. These professions share several characteristics with nuclear emergency responders, including exposure to unpredictable emergencies, high operational demands, and sustained psychological stress. Previous studies have shown that public safety personnel, including firefighters and paramedics, often experience elevated levels of psychological distress due to repeated exposure to traumatic events and demanding work environments (Edgelow et al., 2022; Mackay et al., 2025; Maguen et al., 2025). Therefore, assessing the psychological climate of such organizations may be particularly important for identifying organizational factors that influence mental health and work performance in these high-risk occupational settings. This may help organizations better understand environmental factors that influence psychological wellbeing and operational performance in high-risk professions.

## 6 Conclusion

Although this study completed the Chinese adaptation and reliability and validity testing of the scale, several limitations should be acknowledged.

1. The representativeness of the sample requires improvement. Participants were drawn exclusively from three provinces, resulting in limited geographic coverage. These regions may not fully represent the organizational culture, administrative structures, and operational models of nuclear emergency response teams nationwide. Regional variations in management systems, training intensity, and institutional support mechanisms could influence perceptions of psychological climate. Therefore, the generalizability of these findings to other provinces or organizational settings should be interpreted with caution. Future multi-center studies encompassing broader geographic areas will be conducted to further validate the scale’s structural stability and cross-regional applicability. In addition, male rescue personnel accounted for 97.20% of the sample, with a relatively small proportion of female participants. This extreme gender imbalance limits the ability to assess potential gender differences in perceptions of psychological climate, while also restricting the generalizability of findings to female respondents. Future research will focus on recruiting more gender-balanced samples and conducting measurement invariance tests to examine whether factor structures and psychometric properties remain stable across gender groups.
2. Exploratory and confirmatory factor analyses supported the two-factor structure of the Chinese version of the scale, but further validation is required. This study employed the same sample data for both exploratory and confirmatory factor analyses, which may introduce a risk of bias in model fit results. Previous research indicates that conducting exploratory and confirmatory analyses on identical data samples during scale development may lead to an overestimation of factor structure stability (Worthington and Whittaker, 2006). To enhance robustness, bootstrap sampling was applied 2000 times to assess parameter estimation stability. However, it should be noted that bootstrap methods primarily evaluate parameter stability and cannot fully substitute for structural validation using independent samples. Future research should therefore employ larger, more diverse samples to examine factor structure stability across different occupational roles and organizational contexts. Future research should therefore employ larger, more diverse samples to examine factor structure stability across different occupational roles and organizational contexts and to further evaluate the applicability of the scale to other high-risk occupational groups.

Despite these limitations, this study preliminarily confirms that the Chinese version of the Psychological Climate Scale is a reliable and effective tool for assessing the psychological climate among nuclear emergency personnel. This scale may support future research and organizational management practices aimed at improving the psychological environment and mental health of workers in high-risk emergency response settings.

## Data Availability

The original contributions presented in the study are included in the article/Supplementary material, further inquiries can be directed to the corresponding author.
