# Supplementary material for: Reliability and validity of the Chinese version of the Psychological Climate Scale among nuclear emergency responders
Source: Front Psychol. 2026 Apr 1;17:1805085. doi: 10.3389/fpsyg.2026.1805085 (PMC13079189; doi:10.3389/fpsyg.2026.1805085)
Supplement: Supplementary file 1 [file Table_1.docx]

Supplementary Material

# Chinese version of the Psychological Climate Scale

Q1上级允许我自主决定如何完成工作目标。

Q2上级非常支持我的想法，完成任务的方式及做出的决定。

Q3上级给予我充分的权限，让我能按自己的方式工作。

Q7我的工作职责和需要投入的努力程度都有清晰的说明。

Q8我所在部门的工作标准好理解并可清晰传达。

Q9我在工作中感到自己很有价值

Q10出色地完成本职工作确实创造价值。

Q11我感觉自己是组织里不可或缺的一员。

Q14上级通常很赞赏我的工作方式。

Q15组织认可我所做贡献的重要性。

Q4因为上级常批评新的想法，我不敢轻易提出承担责任。

Q5我相信上级会支持我在工作中做出的决定。

Q6管理层对于我的工作该如何完成，有非常明确的规定。

Q12我的工作对组织非常重要。

Q16我在工作中表达的是真实感受。

Q17在工作中，我能自由地做真实的自己。

Q18在这份工作中表达真实感受是可以的。

Q19我需要全力以赴才能达成工作目标。
